# Schrödinger’s Cheshire Cat: Are Haploid *Emiliania huxleyi* Cells Resistant to Viral Infection or Not?

**DOI:** 10.3390/v9030051

**Published:** 2017-03-18

**Authors:** Gideon J. Mordecai, Frederic Verret, Andrea Highfield, Declan C. Schroeder

**Affiliations:** Marine Biological Association of the UK, Citadel Hill, Plymouth PL1 2PB, UK; gidmord@gmail.com (G.J.M.); fverret@imbb.forth.gr (F.V.); ancba@mba.ac.uk (A.H.)

**Keywords:** *Emiliania huxleyi*, virus, *Phycodnaviridae*, EhV, transcriptome, tiling array

## Abstract

*Emiliania huxleyi* is the main calcite producer on Earth and is routinely infected by a virus (EhV); a double stranded DNA (dsDNA) virus belonging to the family *Phycodnaviridae*. *E. huxleyi* exhibits a haplodiploid life cycle; the calcified diploid stage is non-motile and forms extensive blooms. The haploid phase is a non-calcified biflagellated cell bearing organic scales. Haploid cells are thought to resist infection, through a process deemed the “Cheshire Cat” escape strategy; however, a recent study detected the presence of viral lipids in the same haploid strain. Here we report on the application of an *E. huxleyi* CCMP1516 EhV-86 combined tiling array (TA) that further confirms an EhV infection in the RCC1217 haploid strain, which grew without any signs of cell lysis. Reverse transcription polymerase chain reaction (RT-PCR) and PCR verified the presence of viral RNA in the haploid cells, yet indicated an absence of viral DNA, respectively. These infected cells are an alternative stage of the virus life cycle deemed the haplococcolithovirocell. In this instance, the host is both resistant to and infected by EhV, i.e., the viral transcriptome is present in haploid cells whilst there is no evidence of viral lysis. This superimposed state is reminiscent of Schrödinger’s cat; of being simultaneously both dead and alive.

## 1. Introduction

Coccolithophores are unicellular marine algae that produce a coccosphere made up of calcified platelets commonly referred to as coccoliths [1]. *Emiliania huxleyi* (Haptophyta) is the most ubiquitous and abundant coccolithophorid in modern oceans [2], and forms extensive coastal and mid-oceanic mesoscale blooms at temperate latitudes [3]. Consequently, *E. huxleyi* is a vital contributor to global marine calcium carbonate precipitation and due to the extensive blooms that it produces, plays an important role in the flux of CO_2_ between the atmosphere and the oceans [4,5].

As with most prymnesiophytes, *E. huxleyi* possesses a haplodiplontic life cycle, with distinct heteromorphic differentiation between both ploidy levels, both of which are capable of independent asexual growth [6,7]. The diploid (2N) phase consists of a calcified non-motile cell, whereas the haploid (1N) phase is a non-calcified biflagellated cell bearing organic scales. Blooms of diploid *E. huxleyi* are typically terminated abruptly; releasing coccoliths to the sea surface and organic biomass to the ocean floor, a process which is one of the largest long-term sinks of carbon on earth [5]. Viral infection and subsequent lysis is one of the primary mechanisms for bloom termination and is attributed to the giant, double stranded DNA (dsDNA) *Coccolithovirus*, *Emiliania huxleyi virus* (EhV) [8,9,10].

Previous researchers have shown that haploid *E. huxleyi* cells do not undergo cellular lysis in the presence of EhV, naming this process the Cheshire cat (CC) escape strategy [11], compared to that of the Red Queen (RQ) evolutionary strategy which predicts an “evolutionary arms race” between virus and host [12]. CC dynamics propose that the host can evade infection, and therefore focus resources on interacting with direct ecological competitors [11].

The genome of EhV-86 (407,339 bp), the type species of the genus *Coccolithovirus*, encodes 472 protein coding sequences (CDSs) that were verified through an EhV-86 microarray [10]. The majority of the EhV-86 CDSs have an unknown function; only 66 of 472 (13.9%) were originally annotated with functional protein predictions on the basis of sequence similarity and protein domain matches. More recently the role of some of these protein domains, such as the virally encoded sphingolipid pathway, has been intensely studied [13]. The EhV-86 genome contains a sphingolipid biosynthetic pathway, which previously had never been seen in a viral genome. Glycosphingolipids (GSLs) are part of the building blocks of membrane lipids and lipid rafts and it has been suggested that these genes have undergone horizontal gene transfer between the host [14] and virus [15]. Sphingolipid biosynthesis is implicated in programmed cell death (PCD) [16,17] as it leads to ceramide production, which is an inducer of PCD [18]. Moreover, lytic EhV infection of *E. huxleyi* not only activates the PCD biochemical machinery but also actively recruits and requires GSLs for successful viral replication [19]. In addition, viral GSLs in particular regulate host–virus interactions by inducing host PCD in non-infected cells and facilitating viral production [17].

Virus like particles have been previously described in a haploid *E. huxleyi* dominated bloom [20]. Additionally, a recent study detected novel glycosphingolipids in the haploid *E. huxleyi* RCC1217 strain, which were similar to the viral GSLs observed in the EhV-infected diploid RCC1216 strain; but the authors did not explain the implications of this observation [21]. Although these GSLs are not yet fully characterized, we suggest that haploid cells are not resistant to infection from viruses. To support this hypothesis, we present EhV sequence data obtained via Reverse Transcription-PCR (RT-PCR) as well as the detection of EhV transcripts in a haploid *E. huxleyi* RCC1217 culture by the use of an *E. huxleyi* CCMP1516 EhV-86 combined tiling array (TA). As with the case of Schrödinger’s principle, we suggest that the Cheshire Cat dynamic proposed by Frada et al. [11] is in a superimposed state, in which cells are simultaneously infected and able to avoid lysis.

## 2. Materials and Methods

### 2.1. Strains and Culture Conditions

*E. huxleyi* strain RCC1216 (Table 1) was originally isolated by micropipette isolation of a single diploid cell. The haploid stage of this strain, RCC1217 (Table 1) was isolated after a diploid to haploid shift in a sub-culture of the original strain. These strains were grown in batch cultures in F/2 minus Si medium at 15 °C with a 16:8 light:dark cycle. *E. huxleyi* CCMP1516 (Table S1) was used in EhV-86 infection experiments and to propagate EhV-86 [9], and EhV-86 DNA was used as a positive control for the PCR reaction. The infection dynamic was monitored by flow cytometry as described in Jacquet et al. [22].

### 2.2. Nucleic Acid Extraction

DNA and RNA were extracted from *Emiliania huxleyi* strains (Table 1) when the cultures reached a concentration of at least 10^5^ cells·mL^−1^, during their exponential growth stage. Total genomic DNA was extracted using the DNeasy blood and tissue kit (Qiagen, Valencia, CA, USA) according to the manufacturer’s recommendations. *E. huxleyi* CCMP1516 (50 mL) was inoculated with EhV-86 (50 μL) and DNA was extracted as detailed above at approximately 48 h post infection (p.i.) to use as a control in PCR. DNA was quantified using a NanoDrop 1000 Spectrophotometer (Thermo Scientific, Willington, DE, USA).

Total RNA was extracted using the RNeasy mini-kit (Qiagen), according to the manufacturer’s recommendations, apart from the elution step, in which the RNA was eluted in 50 µL of RNase-free water. The quality of the RNA samples was determined with a Bioanalyzer 2100 (Agilent Technologies, Cheshire, UK). RNA samples were stored at −80 °C.

### 2.3. cDNA Synthesis

To remove contaminating DNA prior to RT-PCR or complementary DNA (cDNA) synthesis, total RNA was treated with RNase-free DNase (Promega, Madison, WI, USA) according to the manufacturer’s instructions. Initially, first-strand cDNA synthesis was performed using the SuperScript III first-strand synthesis system for RT-PCR (Thermo Fisher Scientific, Waltham, MA, USA). Reverse transcription was carried out according to the manufacturer’s instructions using random hexamer primers. The major capsid protein (MCP) gene product was amplified from the Superscript III first strand cDNA.

Double-stranded cDNA transcripts were synthesized using the SMARTer PCR cDNA Synthesis Kit (Clontech, Mountain View, CA, USA), which generates full-length double-stranded cDNA. The kit was used according to the manufacturer’s instructions, apart from an extended incubation time during first strand synthesis (90 min) to create full-length cDNA. The helicase putative viral sequences from the haploid culture were amplified from the cDNA created by the SMARTer PCR cDNA Synthesis Kit.

### 2.4. PCR and RT-PCR Amplification

PCR amplification of the *E. huxleyi* genomic marker coding for a calcium binding protein (CBP) with high Glutamic acid, Proline, and Alanine amino acid content, termed GPA [23], was carried out using the qCBP primers [24] (Table 2). PCR amplification of virus-related genes was undertaken using primers designed to amplify four viral genes: DNA polymerase (DNA pol, ehV030), Helicase (hel, ehv440), proliferating cell nuclear antigen protein (PCNA, ehv440), and the MCP (ehv085) (Table 2).

One-step RT-PCR was used to amplify the haploid specific inner and outer arm dynein heavy chain (DHC) genes using the DHC1b and DHCb primers (Table 2). One-step RT-PCR reactions were carried out in a Rotorgene 6000 QPCR machine (Qiagen). The size of all PCR products was verified by gel electrophoresis on a 1% (*w/v*) agarose gel stained with ethidium bromide in 1× TAE buffer viewed on a UV transilluminator (Syngene, Cambridge, UK).

### 2.5. Sequencing and Alignment

PCR products were purified using the QIAquick PCR purification kit (Qiagen) according to the manufacturer’s recommendations. The purified products were either sequenced directly or cloned using an Invitrogen cloning kit (Thermo Fisher Scientific). Sequencing was performed by Geneservice, Cambridge, UK. The ClustalW function within BioEdit (Ibis Biosciences, Carlsbad, CA. USA) was used for all sequence alignments [25].

### 2.6. Tiling Array

Fifty millilitre subsamples taken from a batch culture of *E. huxleyi* CCMP1516 at a cell density of 5 × 10^5^ cells per mL (C4) were used as negative viral infection controls in our TA experiment. Simultaneously, identical subsample volumes from replicate batch cultures of CCMP1516 (C1) were also taken after an EhV-86 inoculum (final concentration of 1 × 10^6^ viruses per mL) 30 min (S5) and 3.5 h (S6) p.i.; these served as positive viral infection controls. In addition, single extraction time points at cell densities of 5 × 10^5^ cells per mL were taken from *E. huxleyi* RCC1217 (TQ26 1N) and RCC1216 (TQ26 2N) batch cultures. Both of these cultures were not exposed to EhV-86 in this experiment.

RNA extractions were carried out with the RNeasy extraction kit (Qiagen). The first strand complementary RNA (cRNA) Ambion amplification kit (Thermo Fisher Scientific) was used to generate cRNA and the same kit was used in a second step to create double-stranded cDNA. The quality of the RNA samples was verified with a Bioanalyzer 2100 (Agilent Technologies). All these protocols were carried out according to the manufacturer’s recommendations.

The customised TA includes 2,076,726 oligo-nucleotide 50 mer probes spaced by 20 nt on average, designed on one strand of the *E. huxleyi* strain CCMP1516 genome [14]. In addition to *E. huxleyi* probes, the TA includes 18,654 oligo-nucleotide 50 mer probes designed on one strand of the *E. huxleyi* infecting virus EhV-86 genome [10]. A total of 4 two-color fluorescent labelling and hybridisations were carried out by NimbleGen (Table 3). The tiling array hybridization was visualised using SignalMap (NimbleGen, Roche, Madison, WI, USA). The EhV CDSs expressed by RCC1217 and EhV-86 (3.5 h p.i.) (Table S1) were recorded by noting the start and stop of each peak and labelled based on the EhV-86 complete genome annotation available on Genbank [10]. Fluorescence values greater than 3000 (empirically determined when compared to negative controls, RCC1216 and uninfected CCMP1516, expression profiles) was used as a threshold value to determine gene expression. If individual probes were also expressed in CDSs in the negative controls, they were removed and not included. Once the CDSs were recorded, they were compared with previous transcriptional studies [10,28,29]

## 3. Results

### 3.1. Tiling Array

**Figure 1:**
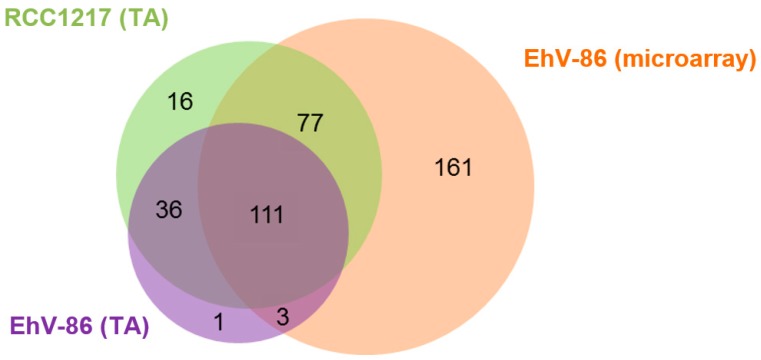
Venn diagram illustrating the comparative Emiliania *huxleyi virus* (EhV) coding sequence (CDS) expression in different strains and between treatments. In green, RCC1217 (1N) complementary cDNA (cDNA) hybridised onto a CCMP1516 + EhV-86 tiling array (TA) (this study). In purple CCMP1516 + EhV-86 3.5 h post infection cDNA hybridised onto the TA (this study). In orange, CCMP1516 EhV-86 cDNA hybridised onto conventional microarrays [10,29].

**Figure 2:**
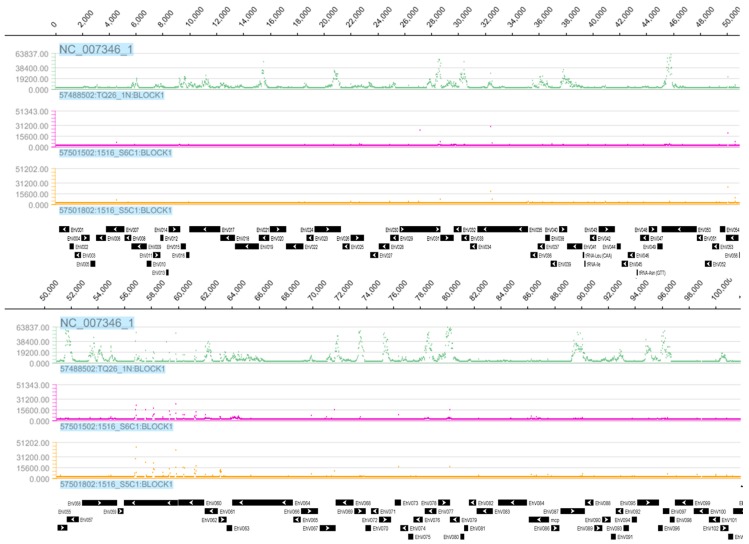

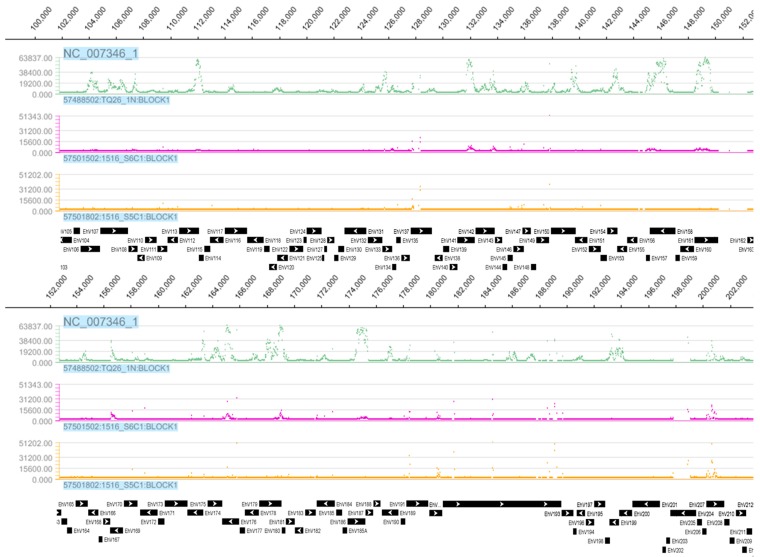

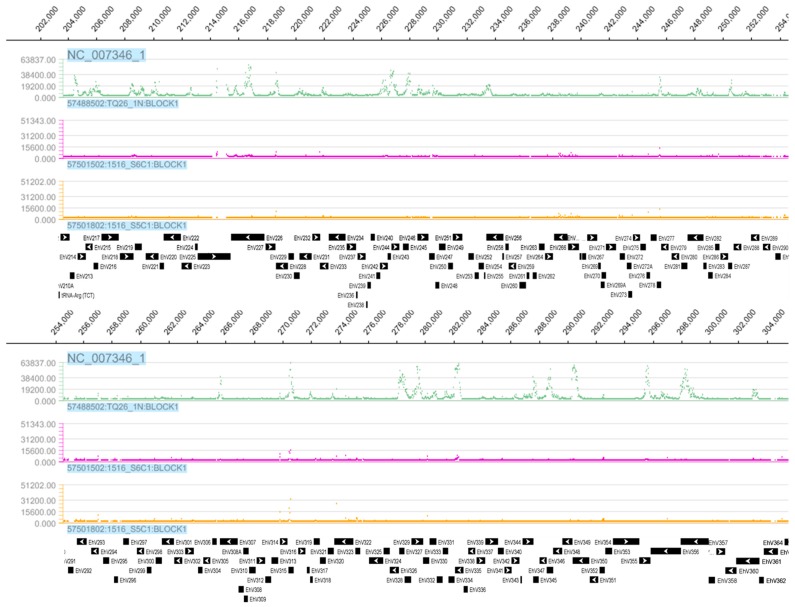

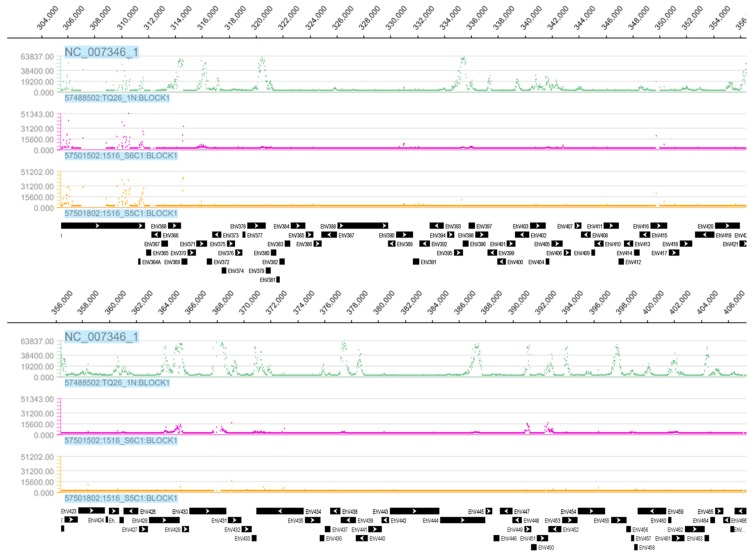
Nimblegen SignalMap displays of fluorescence values from TA for the whole EhV-86 genome (NC_007346_1) in base pairs with corresponding CDS map created by The National Center for Biotechnology Information (NCBI) [31] shown below each set of genome tracks. The first, second, and third tracks in green, purple, and orange display the expression profiles obtained from RCC1217 (1N) from chip 57288502, CCMP1516 infected with EhV-86 3.5 h p.i. from chip 57501502, and CCMP1516 infected with EhV-86 30 min p.i. from chip 57501802 (Table 3), respectively. Individual CDSs are illustrated as solid black rectangles with the internal white arrow head indicating the 5’ to 3’ coding direction.

The EhV-86 TA was used to assess the presence of the EhV transcriptome in RCC1216 (2N), RCC1217 (1N), and CCMP1516 (2N) pre- and post-infection with EhV-86. The CCMP1516 EhV-86 infection dynamic followed the typical culture crash three days post inoculation [9]. The CCMP1516 cell count and EhV-86 virus count were 5 × 10^5^ cells per mL and 0.5 × 10^6^ viruses per mL, respectively, as determined by flow cytometry at 30 min and 3.5 h post infection (data not shown). Tiling arrays allow the identification of novel transcribed sequences, as the arrays are designed to cover the whole genome independent of annotation data. Nonetheless, the expressed regions of the tiling array were labelled according to their location on the EhV-86 genome, using the EhV-86 coding sequence annotation [10]. This enabled the expression profile of the TA data to be compared with previous transcriptional studies (Figure 1, Table S1). Of the total 472 EhV-86 CDSs [10], 389 (82.2%) are expressed during a CCMP1516 EhV-86 lytic infection cycle. No virus expression was detected 30 min p.i. (Figure 2); producing an expression profile identical to an uninfected culture of either CCMP1516 or RCC1216 (data not shown). The CCMP1516 EhV-86 TA data revealed 37 expressed CDSs that were not previously detected, although the expression of two of these (ehv361 and ehv402) have previously been detected using expressed sequence tags ESTs (Table S1).

The EhV-86 TA revealed the strong expression of EhV transcripts in an uninoculated culture of the haploid RCC1217 (Figure 1 and Figure 2, Table S1). The detection of EhV transcripts expressed by the RCC1217 (1N) cells brings the total number expressed up to 85.9% (405 of a total 472). Uninfected cultures of diploid CCMP1516 and RCC1216 confirmed the specificity of the hybridization conditions (Figure 2), only showing small areas of non-specific hybridization, likely due to genes shared between the host and virus which are a result of horizontal gene transfer [30].

The hybridisation profile of the haploid RCC1217 is distinct from that of CCMP1516 infected with EhV-86, indicating that the expression is not a result of EhV-86 cross contamination (Figure 2). In addition, oligo deoxythymine (dT) primed expression assays are susceptible to 3′ bias due to the direction of cDNA synthesis (3′ to 5′) by the enzyme Reverse Transcriptase. The effect of this is seen in the TA hybridisation (Figure 2), in which low probe hybridisation occurred at the 5′ end of the genes. The total number of viral transcripts in the haploid RCC1217 culture (240 CDSs) exceeds that of CCMP1516 + EhV-86, 3.5 h p.i., which expressed a total of 151 CDSs (Figure 1, Table S1). However, the CCMP1516 EhV-86 microarray reported the expression of a total of 352 CDSs at four time points up to 33 h p.i. (Figure 1, Table S1).

Four genes in the EhV-86 genome were identified to be involved in sphingolipid biosynthesis: ehv031, ehv050, ehv077, and ehv079 [10]. All of these were detected in the haploid RCC1217 transcriptome (Table S1). Probe hybridisation of the TA reveals 16 CDSs transcribed by the haploid strain which were not expressed by the CCMP1516 EhV-86 tiling and conventional single probe microarray transcriptomes (Figure 1, Table S1). These 16 CDSs are all unique to the haploid transcriptome apart from ehv093 and ehv415, the expression of which was detected at 24 h p.i. using an EST approach [29]. Only 2 of 16 CDS have a known function assigned to them. Ehv415 is predicted to code for a fatty acid desaturase, a protein involved in membrane lysis, while ehv093 codes for a HNH endonuclease protein, which is involved in DNA homing, restriction, repair, or chromosome degradation [32].

The haploid RCC1217 culture expressed 22 of a possible 25 of the nucleo-cytoplasmic large double-stranded DNA virus (NCLDV) core genes that are present in the EhV-86 genome [33] (Table S2). The haploid transcriptome presents the first recorded expression of the CDS ehv128 by a virus infecting *E. huxleyi* which was not detected in the lytic-phase transcriptional profile of CCMP1516 EhV-86 infection. The CDS ehv128 encodes for a hypothetical ERV1/ALR protein belonging to a large family of proteins that includes the *Saccharomyces cerevisiae* ERV1 (Essential for Respiration and Vegatative growth) protein, which is required for mitochondrial biogenesis, and its homologs in other organisms, the mammalian hematopoetin (alternatively named ALR for its role as an Augmenter of Liver Regeneration), and animal and plant quiescins, so called because of their up-regulation in quiescent cells [34]. The precise functions of these proteins, however, remain unknown. Searches on GenBank on the remainder of the haploid RCC1217 unique transcripts revealed that three of the transcripts contain conserved sequence domains (Table S3), suggesting there are functional viral protein units within the hypothetical protein CDSs expressed by the haploid strain.

The expression of the host sphingolipid biosynthesis pathway was examined using the *E. huxleyi* TA, but no difference was apparent between samples (e.g., Serine palmitoyltransferase (SPT), and Ceramide glycosyltranferase (UGCG), Figure S1). The GSLs detected in *E. huxleyi* are a result of the sphingolipid biosynthesis pathway, which is encoded by both the host and virus as a result of horizontal gene transfer [10,17,35]. Rosenwasser et al. previously demonstrated that these genes are expressed by the host but down regulated upon lytic infection [13]. However, in their experiments down-regulation was observed 24 h p.i., which could explain why our TA expression of this pathway was unresponsive.

### 3.2. E. huxleyi Strain Confirmation

A fragment of the GPA gene, a common molecular marker for genotyping *E. huxleyi* [23], was amplified and sequenced to confirm the identity of the strains. Multiple sequence alignment of the amplified GPA fragment confirmed the identity of the RCC1217 haploid strain in culture (Figure S2).

Additionally, the pronounced transcriptional differentiation between haploid and diploid *E. huxleyi* cells [27] was employed to verify that the haploid culture was indeed in the gametal stage of the haplodiploid life cycle. RT-PCR was used to assess the haploid culture for the presence of 1N specific transcripts. The primer sets DHCb and DHC1b (Table 2) target the cytoplasmic DHC outer arm (OA) and inner arm (IA) genes, respectively, which are linked with a flagella function. The IA and OA flagella genes are solely expressed by haploid *E. huxleyi* cells [27,36], and accordingly were used to validate that RCC1217 was in the haploid form. The IA and OA amplicon sequences amplified from RCC1217 were identical to the previously obtained sequences from the same strain (Figure S3).

In addition to the molecular evidence, light microscopy confirmed the haploid cells to be highly motile (data not shown) and cultures had cells that remained in suspension rather than settling to the bottom as seen with diploid cells. Under normal culturing conditions the RCC1217 culture showed no symptoms of a lytic infection; reaching and maintaining a cell density (typical of a healthy culture) of at least 1 × 10^5^ cells mL^−1^, approximately 7 days post-transfer to fresh media.

### 3.3. Detection of EhV DNA

**Figure 3:**
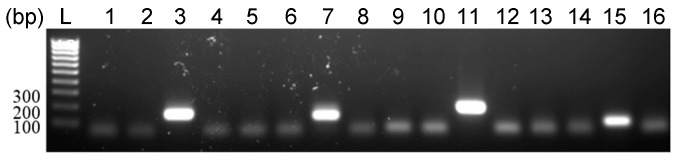
PCR amplification of EhV PCNA (lanes 1–4), MCP (lanes 5–8), helicase (9–12), and DNA polymerase (lanes 13–16) from DNA extracted from RCC1217 (1N) (lanes 1, 5, 9, 13), RCC1216 (lanes 2, 6, 10, 14), and CCMP1516 + EhV-86 (lanes 3, 7, 11, 15). Lanes 4, 8, 12, 16 are negative DNA (no template) controls.

During the lytic infection of EhV, viral DNA is readily amplified by PCR [9]. PCR of four viral genes (PCNA, MCP, helicase, and DNA polymerase) was carried out on DNA extracted from RCC1216, RCC1217, and CCMP1516 infected with EhV-86. Viral DNA was amplified from infected CCMP1516 but not from RCC1216 and RCC1217 (Figure 3). To validate this negative PCR result, a conservative estimate of the sensitivity of the PCR reaction was calculated by amplifying the MCP gene using serial dilutions of the positive control DNA (CCMP1516 + EhV-86 3.5 h p.i). At 3.5 h p.i. the infected CCMP1516 culture are yet to complete crash; however, replication and budding occurs at this stage of the infection [37]. The number of viral particles produced at 3.5 h p.i. was (over-) estimated to be 400, to give a conservative estimate of the equivalent number of viruses that the DNA was extracted from. Positive amplification of the MCP gene was achieved from a 1 in 1,000,000 dilution of the DNA, a volume of DNA equivalent to that extracted from three virions (data not shown).

### 3.4. Detection of EhV RNA

**Figure 4:**
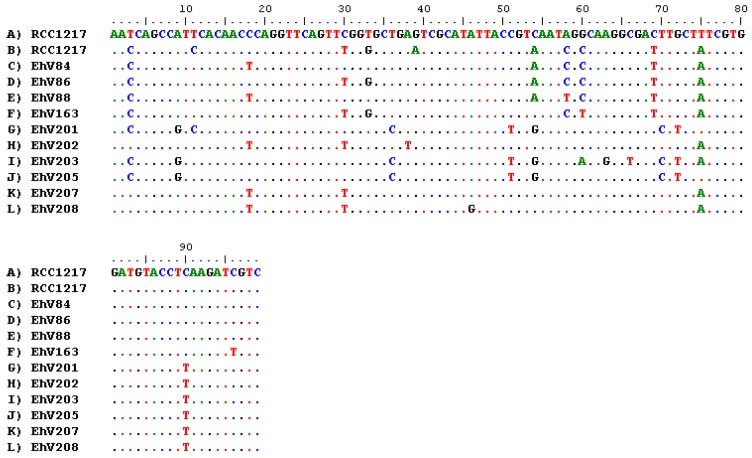
Multiple nucleotide sequence alignment of a 100 bp region of the viral MCP gene (ehv085). The MCP gene was amplified from RCC1217 using reverse transcription polymerase chain reaction (RT-PCR) on two separate occasions (A,B) and in both cases the sequence differed from all previously sequenced MCP genes fragments (C–L) obtained from GenBank [31]. Dots represent positions where the same nucleotides are present as in the top sequence, and letters represent nucleotide substitutions.

**Figure 5:**
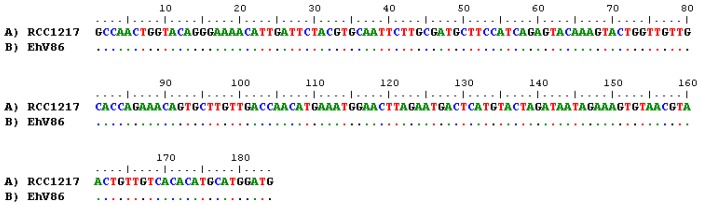
Pairwise nucleotide sequence alignment of a 180 bp fragment of the viral helicase gene (ehv430) amplified using RT-PCR from RCC1217 (1N) aligned with the helicase gene from EhV-86. Dots represent positions where the same nucleotides are present as in the top sequence, and letters represent nucleotide substitutions.

RT-PCR was undertaken on regions of the major capsid protein (ehv085) and helicase (ehv430) genes from RNA extracted from RCC1217 and CCMP1516 infected with EhV-86. MCP-amplified gene fragments from RCC1217 were sequenced, showing homology to the previously characterized genus *Coccolithovirus*, family *Phycodnaviridae* (Figure 4). The MCP sequences obtained on two separate occasions over the period of several years (first amplified in November 2010, then again in April 2012) were different, with 10 nucleotide polymorphisms across the region. However, the substitutions are “silent mutations”, with no change to the resulting amino acid sequence. The helicase amplicon from RCC1217 was identical to the helicase fragment from the EhV-86 genome (Figure 5). Helicase is involved in DNA replication and is consequently a highly conserved gene within the NCLDVs (Table S2).

The presence of viral RNA and hence the state of viral infection is not permanent in the RCC1217 strain. Five years on since the RT amplification in 2012, the RCC1217 strain is virus free (data not shown).

## 4. Discussion

The combination of tiling array data, RT-PCR of amplified NCLDV core genes, sequence data, and the viral-like glycosphingolipids present in the haploid cells as discovered by Hunter et al. [21] strongly indicates that an active EhV infection is present in the haploid *E. huxleyi* RCC1217 cells. To establish whether EhV, a DNA virus, is able to exist solely in an RNA form, we searched the EhV genome for the RNA replicase gene. Viruses with solely RNA-based genomes encode RNA replicase (RNA-dependant RNA polymerase, RdRP), which catalyzes the replication of RNA from an RNA template (the host does not produce an enzyme with this capability). A nucleotide BLAST search of the EhV-86 genome for an RNA replicase nucleotide sequence yields a negative result, implying RNA replicase is absent from the EhV-86 genome. However, EhV-86 codes for six different RNA polymerases (ehv064, ehv105, ehv108, ehv167, ehv399 and ehv434), and it is plausible that one of these or one of the 406 hypothetical proteins could function as an RNA replicase [10].

The majority of the haploid specific transcripts are annotated as hypothetical proteins with an unknown function. Three of these contain conserved protein domains (Table S3). Interestingly, ehv131 contains a calcium binding motif (beta/gamma crystalline), the role of which is unknown, but similarly there are proline rich proteins with calcium binding proteins encoded in the EhV-86 genome in the family B repeat region [38]. Although the function of these calcium binding motifs is not known, they are of interest as calcification in *E. huxleyi* is closely coordinated with cellular metabolism and photosynthesis [39].

The ERV1/ALR protein (ehv128) was the only coding sequence to be solely expressed in the haploid transcriptome (Table S2). The ERV1/ALR protein has been attributed to a cytoplasmic pathway of disulfide bond formation and is thought to represent a class of cellular thiol oxidoreductases [34]. Thiols are a functional group of the amino acid cysteine, and are produced by phytoplankton in response to increased copper concentrations [40]. These organic ligands bind to Cu, reducing free metal concentrations. Copper was shown to disrupt the lytic infection cycle of EhV-86 [26]. Therefore, it would be advantageous for the virus to express genes involved in the thiol biosynthesis pathway, playing a role in a switch from viral persistence to a more acute infection.

The putative haploid RCC1217 virus also expressed a fatty acid desaturase (ehv415) gene and a gene encoding a sterol desaturase conserved protein domain (ehv088) (Table S1), both of which have roles in lipid metabolism. The role of lipids during EhV-86 infection is not well understood, but it is thought that the control of lipid production could be associated with host membrane interactions, which facilitate the intracellular transport and subsequent budding of virus particles [41,42]. The HNH endonuclease family protein (ehv093) (Table S1), a type of restriction endonuclease, was present in the haploid transcriptome. It has been suggested that a virally encoded endonuclease by EhV-86 could lead to the degradation of host DNA [28]. Interestingly, the putative haploid virus expresses a methyltransferase encoding domain (ehv432) (Table S1). In eukaryotes, methylation of DNA bases is involved in epigenetic gene silencing and also plays a defensive role, protecting host DNA against the activity of restriction endonucleases, which cleave foreign DNA as a defense mechanism [43]. A potential function of the virally encoded methyltransferase (ehv452) is to protect viral genetic material from virally encoded endonucleases which degrade host nucleic acids [44].

**Figure 6:**
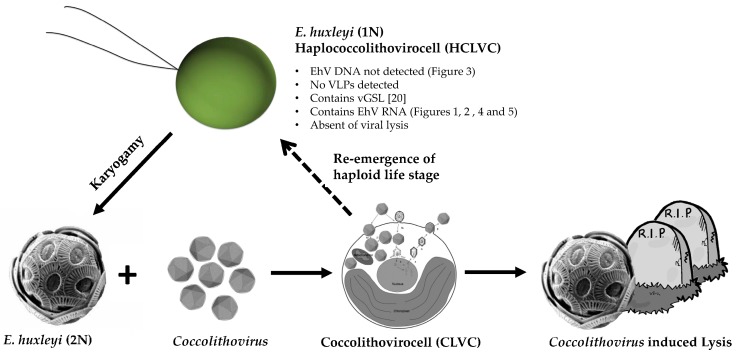
Proposed new *Coccolithovirus*-*E. huxleyi* life cycle incorporating the Haplococcolithovirocell. Infected diploid cells either undergo viral induced lysis or re-emerge as haploid cells containing viral RNA and lipids. Images: CLCV adapted from Mackinder et al. [37], *Coccolithoviruses* adapted from ViralZone [46]. vGSL: viral glycosphingolipids; VLP: virus like particles.

Wilson proposed that the *Coccolithovirus*-*E. huxleyi* pairing (the coccolithovirocell, CLVC) exemplifies this concept due to the unique physiological interactions of EhV and its host [45]. We propose that the viral lipids [21] and EhV transcriptome detected in the haploid infer that haploid cells are a unique part of the *Coccolithovirus* lifecycle, which we propose naming as the Haplococcolithovirocell (HCLVCs) (Figure 6).

Although the viral GSL (vGSL) detected in haploid cells is yet to be fully characterised, judging by the number of EhV transcripts detected in our haploid culture, we propose that these GSLs may contribute to part of the *Coccolithovirus* lifecycle. Viral GSLs are vital for EhV infection as they induce the production of reactive species (oxygen and nitrogen) which initiate programmed cell death. We propose that the HCLVCs help maintain vGSL levels between EhV infection cycles, maintaining an advantage for the virus in the co-evolutionary arms race between virus and host.

Karyogamy (fusion of two haploid cells) has never been observed in haploid *E. huxleyi* cells and we propose that in the case of this culture, RCC1217, this is due to the viral signature in the cells causing a dead end to this stage of the life cycle. However, the RNA viral signature is not permanent; at least not in vitro. Nonetheless, the release of the 1N cell from this putative latent infection state is possible, which in turn would then allow karyogamy to take place. The identity of the vGSL detected in haploid cells is distinct from those found in CLVCs [21]. These differences are consistent with the tiling array data presented here, as the transcriptomic profile of the HCLVCs and CLVCs are distinct.

The majority of giant algal viruses exhibit an acute lytic *r*-selected life strategy characterised by high reproduction rates which eventually lead to cellular lysis. The exception to this rule are the phaeoviruses, which have a K-selected life strategy. Phaeoviruses exhibit viral persistence, and integrate their genomes into the gametal and spore life stages of their hosts, Ectocarpales brown algae. The infected gametes contain a latent provirus, which is then spread throughout the algae during adult development [47]. Overt disease symptoms are seen in the adult reproductive organs, which are deformed and produce virus particles. Although the details of the haploid infecting virus are not identical to the phaeoviruses (specifically, the absence of viral DNA), there are some interesting analogies, namely the switch to viral latency in the gametal life stages. We propose that, similarly to phaeoviruses, coccolithoviruses also have a secondary life cycle strategy in gametal *E. huxleyi* cells. However, there is no evidence of genome integration as EhV DNA was not detected in the haploid cells. We suggest that the EhV transcriptome is present in haploid cells in a novel mechanism of viral persistence.

Viruses infecting *E. huxleyi* exist in a world of two halves; during a bloom, host abundance is extremely high, and there are plenty of opportunities for re-infection and horizontal transmission. Conversely, between blooms host abundance is lower and the viral decay rate is high [48], and as a result there is less opportunity for horizontal transmission. We propose that within coccolithoviruses, two distinct infection strategies exist, which occupy different ecological niches and each is suited to the boom and bust ecology of the host. The infection of the haploid cells by an RNA viral life stage may help to explain the stability of the coexistence of virus and host in marine algae.

Flow cytometric analysis has shown the emergence of a sub-population of haploid cells after virus-mediated bloom demise [22]. Additionally, gene expression studies found that infection by EhV increased expression of pathways related to spermatogenesis [13], suggesting that the virus initiates gametogenesis and is an integral part of the *E. huxleyi* lifecycle (Figure 6). Previous work used PCR to show that EhV does not replicate in haploid *E. huxleyi* cells [11]. In HCLVCs we have detected the absence of EhV DNA, but the presence of RNA. The mechanisms behind this remain unexplored and there is no other example of a DNA virus with a separate RNA life stage where DNA is not detectable. The data presented here in combination with the viral GSLs detected in haploid cells by Hunter et al. [21] depict a different scenario to the Cheshire Cat dynamic. Future metabolomic and transcriptional studies will hopefully ascertain the breadth and mechanism of this infection.

## 5. Conclusions

Coccolithoviruses drive *E. huxleyi* bloom dynamics and therefore play an integral role in the global ocean carbon pump and climate [45]. The realisation that the host-virus dynamic is not always lytic and that covert infection can arise, highlights our limited understanding into the full extent in which they control their host. This study clearly demonstrates the potential of NCDLVs to interact with and manipulate the ecology of their hosts; the life cycle of which is not fully understood for both the host and their viruses. Although persistence has been revealed in other phycodnaviruses [47], the potential mechanism seen in haploid *E. huxleyi* cells, in which solely the viral transcriptome is detected in gametes, is entirely novel. Despite our limited knowledge, coccolithoviruses are clearly extraordinary viruses which deserve further research.

## Figures and Tables

**Table 1:** Host and virus strains used in the current study.

Strain	Origin	Date of Isolation
*Emiliania huxleyi* RCC1216 (TQ26 2N)	Tasman Sea	October 1998
*E. huxleyi* RCC1217 (TQ26 1N)	from strain *E. huxleyi* RCC1216	July 1999
*E. huxleyi* CCMP1516	South Pacific	July 1991
*E. huxleyi* Virus 86	English Channel [9]	July 1999

1N: haploid; 2N: diploid.

**Table 2:** Primers used in this study.

Primer	Sequence (5′ to 3′)	Target (CDS)	Ta (°C)	Reference
qCBP_F	AGTCTCTCGACGCTGCCTC	GPA	60	[24]
qCBP_R	TGGCCTAGCACCAGTCTTTGG			
MCP_F2	TTCGCGCTCGAGTCGATC	MCP (ehv085)	60	[8]
MCP_R2	GACCTTTAGGCCAGGGAG			
EhVhel_F	GCCAACTGGTACAGGGAAAA	Helicase (ehv104)	54	[26]
EhVhel_R	CATCCATGCATGTGTCACAA			
EhVPCNA_F	GGGCATTTCATTTGCCATAC	PCNA (ehv440)	54	[26]
EhVPCNA_R	ATTCTCCGTCGACAAACGC			
EhVpol_F	TATAATGCACGCCAACTTGC	DNA pol (ehv030)	54	[26]
EhVpol_R	GCAATTGCACCAAGTGGATA			
DHC1b_F	GCTTTCTCACTGCGCTCAT	Flagellar inner DHC	55	[27]
DHC1b_R	GTAGAGCGGGCACGAGTACA			
DHCb_F	TGAACCTCGTCCTCAACACA	Flagellar outer DHC	55	[27]
DHCb_R	GAATCATCGGCATCACTGG			

CDS: coding sequences; DHC: dynein heavy chain protein; GPA: glutamic acid, proline and alanine rich protein; MCP: major capsid protein; PCNA: proliferating cell nuclear antigen protein.

**Table 3:** Tiling Array hybridization design (081216_Ehux_DS_CGH_HX1) carried out by NimbleGen (design ID 8739, ORD_ID 29312).

CHIP_ID	Image Name	Dye	Sample Description *	Virus Inoculation
57501502	57501502_532.tif	Cy3	1516 S6C1	3.5 h p.i.
	57501502_635.tif	Cy5	1516 S5C1	0.5 h p.i.
57501802	57501802_532.tif	Cy3	1516 S5C1	0.5 h p.i.
	57501802_635.tif	Cy5	1516 S5C4	No
58219802	58219802_532.tif	Cy3	1516 S6C4	No
	58219802_635.tif	Cy5	1516 S6C1	3.5 h p.i.
57488502	57488502_532.tif	Cy3	TQ26 1N	No
	57488502_635.tif	Cy5	TQ26 2N	No

*: S indicates the sample time point, while C indicates the culture replicate; p.i.: post infection.

## Supplementary Materials

The following are available online at www.mdpi.com/1999-4915/9/3/51/s1, Figure S1: CCMP1516 tiling array, Figure S2: Multiple sequence alignment of PCR-amplified GPA sequence from RCC1217 (1N) compared to previously sequenced GPA amplicons, Figure S3: Pairwise nucleotide sequence alignment of fragments of the amplified RT-PCR products of the dynein heavy chain (DHC) (A,B) inner arm (DHC1b) and (C,D) outer arm (DHCb) genes, Table S1: Unique EhV-86 CDSs expression data, Table S2: Presence of NCLDV Core genes in the EhV-86 genome and in the transcriptome of the virus infecting RCC1217, Table S3: Haploid unique transcripts in the EhV-86 genome and in the transcriptome of the virus infecting RCC1217.
