# Supplementary material for: Schrödinger’s Cheshire Cat: Are Haploid Emiliania huxleyi Cells Resistant to Viral Infection or Not?
Source: Viruses. 2017 Mar 18;9(3):51. doi: 10.3390/v9030051 (PMC5371806; doi:10.3390/v9030051)
Supplement: Supplementary file 1 [file viruses-09-00051-s001.pdf]

Supplementary Materials: Schrödinger's Cheshire Cat: Are Haploid *Emiliania huxleyi* Cells Resistant to Viral Infection or Not?

Gideon J. Mordecai, Frederic Verret, Andrea Highfield and Declan C. Schroeder

Table S1. Unique EhV-86 CDSs expression data. As detected by (**A**) RCC1217 (this study), (**B**) CCMP1516+ Ehv-86 3.5 h p.i (this study), (**C**) EhV-86 microarray 1, 2, 4 and 33 h p.i. (Wilson et al 2005 and Allen et al 2007) and (**D**) no study. CDS (bold) show expression detected by the EhV-86 EST library 24 h p.i. (Kegel et al 2010). CDS (underlined) are those with a predicted functional protein.

**A**	**B**	**A, B**	**C**	**A, B, C**	**A, C**	**B, C**	**D**
012, 049, 067, 088, **093**, 125, 128, 131, 137, 171, 195, 259, 375, **415**, 432, 457	060	032, 033, 035, 043, 056, 069, 078, 092, 096, 102, 114, 117, 157, 168, 178, 189, 222, 225, 248, 255, 287, 318, 321, 327, 329, 333, 345, 347, 356, **361**, 371, 396, 398, **402**, 433, 458	001, 002, 003, 005, 007, 009, 013, **014**, 021, 022, **023**, 025, **028**, 044, 047, 051, 052, 053, 054, 059, 065, 070, 071, 075, 080, 083, 084, 086, 089, 094, 099, 100, 101, **103**, 104, **105**, 109, 118, 119, 122, 123, 127, 129, 130, 135, 138, 139, 140, 148, 149, 156, 162, 163, 164, 167, 172, 173, 175, 177, 181, 182, 183, 185, 185A, 187, 188, 190, 191, 197, 201, 204, 205, 206, 209, 210A, 211, 212, 217, 221, 232, 234, 237, 250, 252, 260, 265, 266, 267, 268, 270, 271, 272A, 274, 275, 277, 280, 281, 284, 286, 289, 290, 291, 292, 293, 294, 295, 296, 297, 298, 300, 302, 303, 304, 305, 306, 308, 308A, 311, 312, 313, 314, 315, 316, 320, 322, 323, 336, 337, 338, 351, 352, 353, 354, 359, 360, 369, 372, 380, 382, 384, 385, 388, 391, 392, 406, 408, 410, 411, 412, 413, 414, 417, 420, 427, 435, 442, 445, 447, 448, 463, 467	015, 019, **020**, 024, 029, **030**, *031*, 037, 040, **041**, *050*, 057, 058, **061**, 062, 063, **064**, 068, 072, *077*, *079*, 087, 095, 097, 098, 106, 107, **108**, 112, 113, 132, **133**, 134, 141, 142, 143, 151, 154, **158**, 160, 161, 165, 169, 174, 176, **179**, 186, 192, 199, 200, 213, 216, 218, 220, 223, 226, 227, 228, 230, 241, 242, 243, 244, 245, 246, 247, 256, 278, 279, 288, 307, 328, 334, 335, 350, 355, 357, 370, 373, 378, 379, 394, 395, **397**, **399**, 403, 404, 405, 418, 419, 421, 424, 425, **428**, 429, **430**, **431**, **434**, 436, 437, 439, **440**, **444**, 449, 450, 452, 453, **455**, **459**, 461, 464	004, 006, 011, 016, 017, **018**, **026**, 027, 036, 038, 048, 066, 074, 076, 082, 090, 091, **110**, 115, 120, 121, 124, **136**, 144, 146, 147, 150, **152**, 153, 155, 170, **184**, 198, 208, 210, 214, 215, 219, 231, 233, 235, 249, 251, 253, 254, 263, 264, 269A 282, 301, 330, 331, 332, 339, 342, 344, 346, 348, **349**, 367, 368, 387, 393, 401, 407, 422, 423, 426, 438, 446, **451**, 454, 456, 462, 465, 466, 468	008, **085**, 229	010, 034, 039, 042, 045, 046, 055, 073, 081, 111, 116, 145, 159, **166**, 180, 193, 194, 196, 202, 203, 207, 224, 236, 238, 239, 240, 257, 258, 261, 262, 269, 272, 273, 276, 283, 285, 299, 309, 310, 317, 319, 324, 325, 326, 340, 341, 343, 358, 362, 363, 364, 365, 366, 374, 376, 377, 381, 383, 386, 389, 390, 400, 409, 416, 441, 443, 460

CDS (italised) are those involved in the sphingolipid pathway identified by Wilson et al. 2005.

Table S2 Presence of NCLDV Core genes in the EhV-86 genome and in the transcriptome of the virus infecting RCC1217.

Function	EhV-86 CDS	RCC1217
Vaccinia Virus D5-type ATPase	ehv459	+
DNA Polymerase	ehv030	+
Vaccinia Virus A32-Type ATPase	ehv072	+
Vaccinia Virus A18-Type Helicase	ehv104	
Capsid Protein	ehv085	+ *
Thiol-oxidoreductase	ehv128	+
Vaccinia Virus D6R-Type Helicase	ehv141	+
Ser/Thr Protein Kinase	ehv141	+
VLTF2-Like Transcription Factor	ehv438	+
TFII-Like Transcription Factor	ehv105	
MuT-Like NTP Pyrophosphohydrolase	ehv398	+
Proliferating Cell Nuclear Antigen	ehv020	+
Ribonucleotide Reductase, Large Subunit	ehv428	+
Ribonucleotide Reductase, Small Subunit	ehv026	+
Thymidylate Kinase	ehv431	+
dUTPase	ehv397	+
A494R-Like Uncharacterised Protein	ehv403	+
Capping Enzyme	ehv453	+
ATP-Dependent Ligase	ehv158	+
RNA Polymerase, Subunit 1	ehv064	+
RNA Polymerase, Subunit 2	ehv434	+
Thioredoxin/ Glutaredoxin	ehv465	+
BIR Domain	ehv166	+
Topoisomerase II	ehv166	+
RNA Polymerase, Subunit 10	ehv167	

* Amplified by RT-PCR

Table S3. Haploid unique transcripts containing conserved protein domain homologues identified through BLASTx (20.8.12).

CDS	Conserved Domain	Function / comments
ehv088	ERG3	Sterol desaturase (lipid metabolism)
ehv131	Crystall	Beta/gamma crystalline, a universal motif for calcium binding
ehv432	AdoMEt_MTases superfamily	Methyltransferase encoding domain

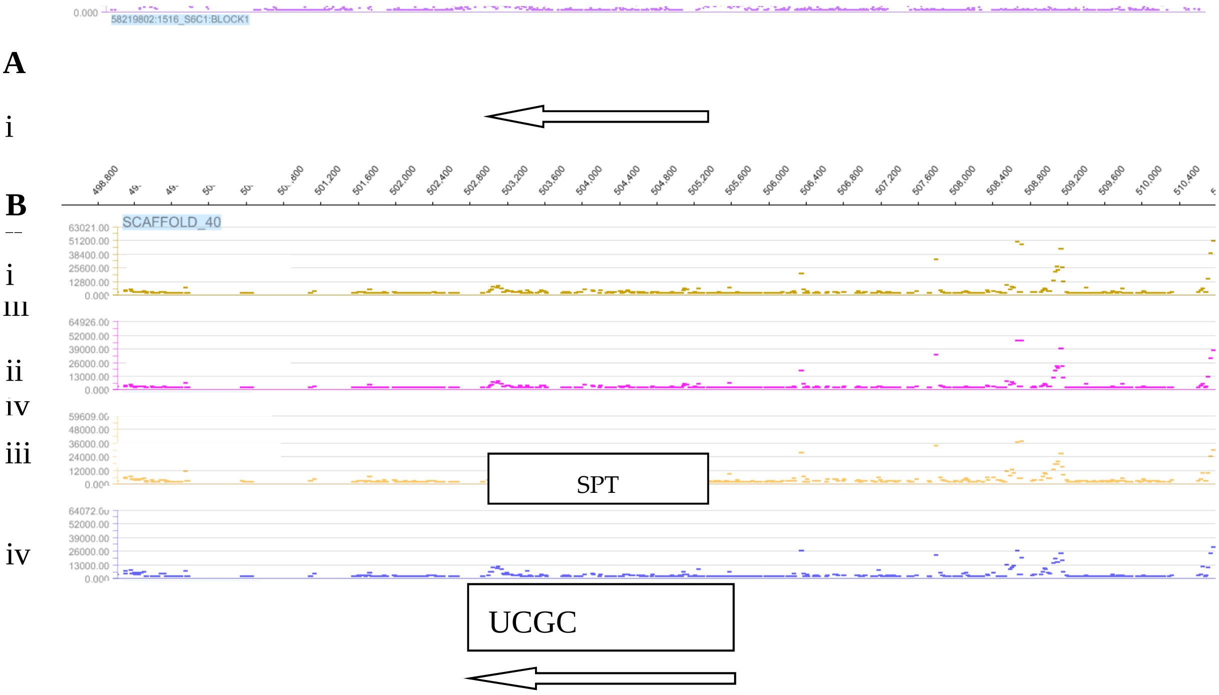

Figure S1. CCMP1516 tiling array. Shows expression of (**A**) Serine Palmitoyl Transferase (SPT): scaffold_7: 1,016,211-1,020,940 bp; and (**B**) Ceramide glycosyltransferase (UCGC): scaffold_40: 503,190-505,637 bp highlighting gene position (boxed) with the 5′ to 3′ direction shown by arrows annotated below. The 50 bp probes (data points) span the whole EhV-86 genome (x axis), the height of the fluorescence signal (y axis) illustrates the relative expression of each probe. The TA shows the expression of viral transcripts when i) RCC1217 (1N) is labeled with Cy3 on chip 57288502; ii) RCC1216 (2N) is labeled with Cy5 on chip 57488502; iii) CCMP1516 infected with EhV-86 3.5 hours p.i. is labeled with Cy3 or Cy5 on chips 57501502 or 5819802, respectively; iv) CCMP1516 (uninfected) or 30 min p.i. labeled with either Cy dyes on various chips.

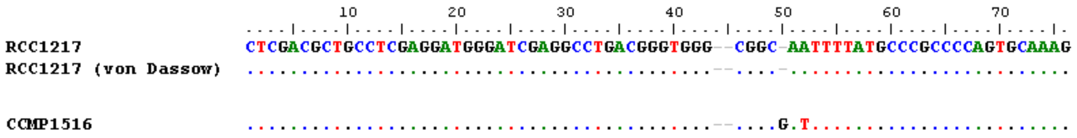

Figure S2. Multiple sequence alignment of PCR-amplified GPA sequence from RCC1217 (1N) compared to previously sequenced GPA amplicons. Previous amplicons are: RCC1217 (Von Dassow et al 2009) and CCMP1516 (Schroeder et al 2005). Dots represent identical nucleotides compared to the top sequence, letters represent nucleotide substitutions and dashes represent insertion/deletion

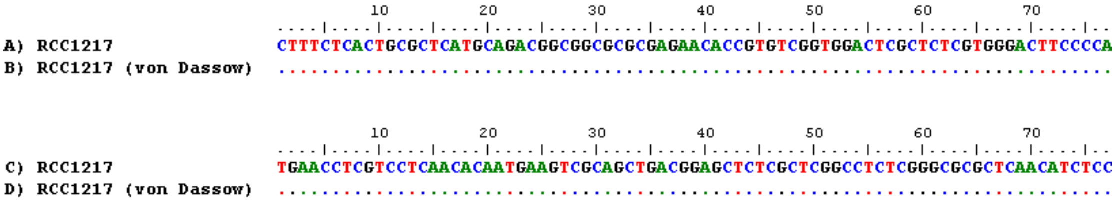

Figure S3. Pairwise nucleotide sequence alignment of fragments of the amplified RT-PCR products of the dynein heavy chain (DHC) (**A**,**B**) inner arm (DHC1b) and (**C**,**D**) outer arm (DHCb) genes. Dots represent positions where the same nucleotides are present as in the top sequence. The sequences amplified from RCC1217 (A,C) are aligned with previously obtained sequences from strain RCC1217 (B,D) (Von Dassow et al. 2009).

© 2017 by the authors. Submitted for possible open access publication under the terms and conditions of the Creative Commons Attribution (CC-BY) license (http://creativecommons.org/licenses/by/4.0/).
